# Optimising the quality of multidisciplinary team meetings: A narrative review

**DOI:** 10.1002/cam4.4432

**Published:** 2022-03-07

**Authors:** Thanh Hai Tran, Jasper de Boer, David E. Gyorki, Meinir Krishnasamy

**Affiliations:** ^1^ University of Melbourne Melbourne Victoria Australia; ^2^ Peter MacCallum Cancer Centre Melbourne Victoria Australia; ^3^ Department of Surgery University of Melbourne Melbourne Victoria Australia; ^4^ Victorian Comprehensive Cancer Centre Melbourne Victoria Australia; ^5^ Sir Peter MacCallum Department of Oncology University of Melbourne Melbourne Victoria Australia; ^6^ Division of Cancer Surgery Peter MacCallum Cancer Centre Melbourne Victoria Australia

**Keywords:** cancer, cancer multidisciplinary team meetings, decision‐making, patient care team, teamwork

## Abstract

**Background:**

Understanding of factors that contribute to implementation of effective cancer multidisciplinary team meetings (MDMs) is still limited. Published literature on the effect of teamwork function, leadership roles, decision‐making processes and structural components on the quality of MDMs was reviewed and synthesised.

**Methods:**

In this paper, a MEDLINE review (September 2020) was performed to assess clinical decision‐making in the context of MDM discussions.

**Results:**

Twenty‐nine eligible studies were included. Six studies addressed the infrastructural aspects of MDMs. Nine studies used either qualitative or mixed method approach to develop and validate observational tools to assess the quality of MDMs. Seven studies used qualitative approaches to explore the opinions of MDM members on factors that impact on the effectiveness of MDMs. Five studies used validated observational tools to observe and assess the effectiveness of MDMs. One prospective study explored the relationship between quality of information presented at MDMs and ability of MDM members to make clinical decisions. The final study prospectively tested the ability of a multicomponent intervention to improve decision‐making processes within MDMs.

**Conclusions:**

A broad range of factors including teamwork, leadership, case complexity, decision‐making processes and availability of patient information were identified to impact the quality of MDMs. Evidence currently available largely focuses on the development of tools to identify factors in need of improvement to optimise MDMs. Robust research is required to identify the factors that are demonstrated to enhance MDM quality which can then aid the standardisation of how MDMs are conducted.

## Graphical abstract

This review highlights how multidisciplinary meetings function to generate clinical recommendation on a treatment plan. We highlight that further research is needed to assess the efficiency and effectiveness of multidisciplinary team meetings to improve the workflow for clinicians and outcomes for patients.

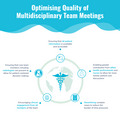

## 1 INTRODUCTION

Cancer is a leading cause of health burden worldwide, associated with high rates of morbidity and mortality.^1^ With the increasing complexity of cancer diagnosis and treatment, optimal management of cancer patients requires the input of a multidisciplinary team (MDT)^2^ and cancer MDTs or tumour board (MTB) meetings have become an essential component of cancer care delivery globally.^3^ The purpose of multidisciplinary team meetings (MDMs) is to provide opportunity for health professionals with differing expertise in diagnosing, managing and caring for people with cancer to meet on a regular basis to generate treatment recommendations based on best evidence whilst taking into consideration patient preference.^4^, ^5^

Whilst MDMs play a central role in cancer management, there is limited evidence to support their effectiveness.^2^ Currently there is poor understanding of what constitutes an effective MDM and consequently, uncertainty about how best to measure the effectiveness. Evidence of the impact of MDMs on patient outcomes is equivocal^6^ and, despite some evidence of survival benefits for colorectal, oesophageal, sarcoma and other cancer patients,^7^, ^8^, ^9^ it remains unclear which, if any component of an MDM specifically impacts patient outcomes. In recent years, research attention has shifted from assessment of MDM impact on survival outcomes to the structure and functioning of MDMs,^10^ with particular focus on the quality of teamwork, leadership and decision‐making processes as measures of effectiveness.^11^

The aim of this narrative review was to explore factors proven to be associated with the effective implementation of cancer MDMs. The research question was: What are the proven teamwork, infrastructural and logistical factors necessary for an effective cancer MDM and how can such factors be integrated into a standardised framework to inform the quality of MDMs.

## 2 METHODOLOGY

### 2.1 Search strategy and selection criteria

A comprehensive literature search was conducted in August and September 2020, using Medline (Ovid) as a more focused search engine, to identify relevant papers. The following Medical Subject Headings (MeSH) and keywords were used in the search: (patient care team OR multidisciplinary team) AND (interdisciplinary communication OR leadership OR decision‐making) AND (structure OR logistic OR infrastructure OR organisation OR framework OR guideline) AND (quality improvement OR effectiveness OR efficiency OR efficacy) AND (medical oncology OR neoplasms) AND (tumour board OR meeting). Full search strategy terms are listed in Data S1. Additional studies were selected independently from the reference lists of included papers. Results were limited to papers published in the English language, empirical studies including qualitative, quantitative and mixed method study designs and studies that focused on cancer MDMs. Papers were included if they addressed factors that impact on the quality of MDMs such as organisation, information availability, teamwork, leadership and decision‐making processes.

### 2.2 Risk of bias

Due to the heterogeneity of study designs it was not feasible to conduct a risk of bias assessment on included studies.

## 3 RESULTS

### 3.1 Study characteristics

**FIGURE 1:**
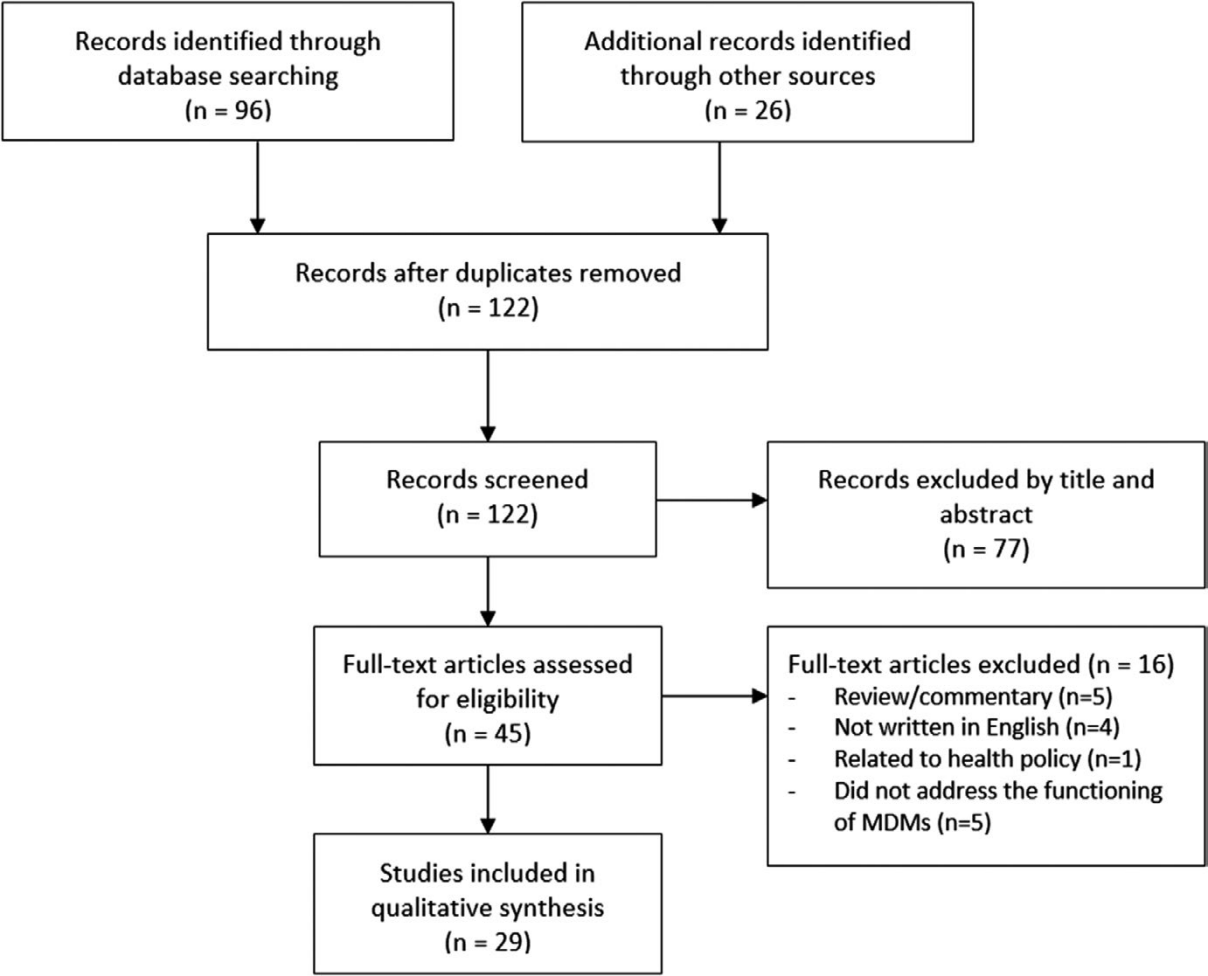
PRISMA flow chart

The search strategy identified a total of 122 citations (Figure 1). All citations were screened by the review author (HT). Of the 29 eligible papers, the majority were qualitative studies or mixed method studies, with one study by Lamb et al^12^ being a prospective interventional study. Characteristics and key findings are illustrated in Table S1. Although it was not possible to undertake a meaningful risk of bias assessment, a limitations column has been included in Table S1 to highlight some potential biases and weaknesses within each study.

### 3.2 The optimal structure of MDMs

Two studies attempted to use literature available to develop a quality criteria guideline for effective MDMs.^10^, ^13^ Drawing on a pre‐existing guideline called ‘The Characteristics of an effective MDT’, developed from a UK national survey completed by over 2000 MDT members,^14^ one study used 18 of the 86 characteristics to develop the MDT Observational Assessment Rating Scale (MDT‐OARS).^13^ The scale applied criteria for quality assessment across four main domains relating to teamwork, clinical decision‐making, the infrastructure for meetings and the meeting organisation and logistics. In another study, Ottevanger et al^10^ developed a guideline based on a literature search and a consensus‐based statement from an expert panel. The quality criteria for this guideline were characterised into four broad categories relating to the organisation of the meeting, the core members and their responsibilities, the meeting itself and the documentation of the meeting recommendations. There were many commonalities in the quality criteria across both guidelines including: attendance of all core team members (surgical oncologist, medical oncologist, radiation oncologist, pathologist and radiologist), the role of the chairperson, the communication between team members, as well as the preparation of patient notes. In terms of the MDM decision‐making processes, patient‐centred care was included as a criterion in the guideline by Taylor et al,^13^ whilst the guideline by Ottevanger et al^10^ had an additional focus on the documentation of decisions made at MDMs. The importance of these guidelines was emphasised by an observational study by Soukup et al noting that more than half the case reviews take place between only two or three disciplines in the meetings.^15^

One study explored the role of streamlining patient discussions as opposed to discussing every patient.^16^ The authors defined streamlining discussion as enabling the prioritisation of more complex cases during MDMs over straightforward cases, which can be discussed briefly, or excluded from MDMs altogether. The rationale appears to be related to reducing the burden of time pressures and excessive case loads in order to improve the effectiveness and efficiency of MDMs.^12^, ^17^

In a qualitative study exploring the impact of an oncology nurse navigator (ONN) on physician adherence to guidelines and streamlining patient care in the setting of a lung cancer MDM,^18^ authors concluded that ONNs can play a central role in MDMs by creating and implementing standardised practice pathways. These in turn may improve adherence to national guidelines. Furthermore, this study evaluated the role of ONNs in communicating information discussed at MDMs with the patient to better coordinate care.

The impact of radiologist involvement in MDMs was explored in a study by Neri et al.^19^ As suggested in the guidelines developed by Ottevanger et al^10^ and Taylor et al,^13^ the attendance of radiologists as core members of MDMs, is an important quality criteria, with findings from a survey of 292 radiologists from the European Society of Oncologic Imaging, reporting that their attendance at MDMs was beneficial in changing diagnostic strategies and refining therapeutic decisions made. Despite this knowledge, Neri et al^19^ noted that radiologists were not mandated to routinely attend MDMs.

### 3.3 Outcome measures of an effective MDM

The quality of the decision‐making process was a common outcome measure in many of the included studies. Lamb et al focused heavily on the evaluation of factors affecting decision‐making processes in MDMs.^12^, ^20^, ^21^, ^22^ In one study, Lamb et al, concluded that the availability of radiological (*r *= 0.23, *p* ≤ 0.01) and pathological information (*r *= 0.39, *p* ≤ 0.01) were associated with higher rates of decision‐making.^22^, ^23^ Importantly, information on patient's co‐morbidities and psychosocial history was found to be lacking at many MDMs.^5^, ^22^, ^24^ This was identified as a common barrier to reaching a clinical decision.^2^, ^17^ Furthermore, the ability to implement the clinical decisions made was another factor that affected the quality of MDMs.^17^, ^25^ Implementation of MDM recommendations and adherence to national guidelines were audited by Ghazal Asswad et al. Retrospective analysis of patient records showed that, in most cases, MDM recommendations were implemented and adhered to national guidelines. However, cases of discordance between MDM recommendations and adherence to guidelines were found to be clinically justifiable.^25^

Team functioning and performance were found to be important drivers of effective clinical decisions in MDMs. In one mixed methods study observing lung cancer MDMs, it was identified that there was limited communication between doctors, nurses and allied health professionals.^26^ Additionally, discussions were heavily dominated by doctors, with little input from nurses and allied health professionals.^20^, ^26^ Soukup et al^23^ found that when a doctor, commonly a surgeon, took on the role of the chairperson whilst also having responsibility to provide input regarding treatment recommendations, coordination of meetings was negatively affected, which impacted on overall team functioning.^23^, ^27^ The need for optimal structure of MDMs with clear role assignments becomes even more apparent for national virtual MDMs. Rosell et al noted an increase in barriers that prevent optimal decision‐making including uncertain assignments and responsibilities, suboptimal collaboration between hospitals and non‐attendance from core members.^28^

### 3.4 Development of assessment tools for MDMs

Seven studies used either a qualitative or mixed method approach to develop and validate an observational tool to assess the quality of MDMs. One tool was the MDT Metric of Decision‐Making (MDT‐MODe) which measures the quality of presented patient information, contributions of MDT members based on specialty and a team’s ability to make a clinical decision.^29^ This tool was adapted by Lumenta et al^11^ and Hahleg et al^24^ in their respective studies to assess the quality of teamwork and decision‐making in MDMs.

Lamb et al developed the MDT Quality Improvement Checklist (MDT‐QuIC) which was designed to ensure that clinical decision‐making is comprehensive, holistic and patient‐centred.^21^ They incorporated the MDT‐QuIC into a multi‐component intervention which involved training MDT members on the principles and evidence of effective clinical decision‐making, and used the MDT‐QuIC to assess and provide written guidance on decision‐making for the MDT members.^12^ This prospective interventional study reviewed 36 urological MDMs and showed improvements in the number of decisions made, global information quality and global teamwork scores when the tool was used.

Patkar et al., developed a novel clinical computerised decision support platform (the Multidisciplinary meeting Assistant and Treatment sElector‐MATE), to generate patient‐specific treatment decisions recommendations based on diagnostic markers, histopathological data, patient co‐morbidities and evidence‐driven guidelines uploaded onto the MATE database.^30^ When comparing the MATE recommendations to the actual MDT decisions made in 1058 breast cancer cases, the level of concordance between clinician and computer enabled decisions was 93.2%, suggesting a potential role for such platform at MDMs. However associated costs, scalability, the ability for the platform to store large volumes of clinical evidence and its transferability to other cancer MDMs were noted as limitations to its broad implementation.^30^ Similarly, Pluyter et al developed a clinical decision support system (CDSS) to aid the decision‐making process in lung cancer MDMs.^31^ The CDSS was designed to present relevant clinical data in a practical and easily accessible manner. The CDSS was shown to support the team in ensuring accurate diagnosis and TNM classification. The system also allowed for cross‐validation of diagnostic findings, identified discordance between diagnostic tests, facilitated cancer staging according to diagnostic evidence and alerted contra‐indications for personalised treatment recommendations. Although there is potential for use of CDSS to aid clinical decision‐making at MDMs, this study only tested the function of CDSS in a single‐simulated MDM discussing eight primary lung cancer cases. Additional research is required to test the function of CDSSs in different MDMs and in a larger sample of cases before they can be implemented.^31^

Tools developed that specifically assessed the quality of teamwork during MDMs were reported in several studies. Taylor et al developed two tools: the MDT Observational Assessment Rating Scale (MDT‐OARS)^13^ and the Team Evaluation and Assessment Measure (TEAM).^32^ The MDT‐OARS assesses 18 elements of good team functioning based on the 86 items in ‘The characteristics of an effective MDT’.^14^ Alternatively, the TEAM is a self‐assessment tool developed for MDT members to evaluate their own team functioning and identify areas for improvement. This tool was reported to be acceptable in a pilot study of 10 bowel cancer MDMs, in terms of feasibility and inter‐rater reliability scores. Further adapting the TEAM tool, Harris et al first developed a prototype observational tool^33^ which was later modified to become the MDT Meeting Observational Tool (MDT‐MOT).^34^ This tool assesses team attendance, leadership and chairing of MDMs, teamwork and culture. The intention was for the tool to be used routinely at MDMs by clinical and non‐clinical professionals to highlight areas for improvement and support team development.

Case complexity affects the capacity of MDT members to make a clinical decision.^22^ To assess the complexity of a cancer patient's case and facilitate the streamlining of patient cases, Soukup et al^35^ developed a novel tool called the Measure of case‐Discussion Complexity (MeDiC) tool. This tool was developed to standardise the case selection process for MDMs by identifying complex cases which can be prioritised for discussion. It was proposed that less complex patient cases identified by this tool may not necessarily need to be discussed at MDMs, if well‐defined guidelines are available to guide decision‐making.^35^

## 4 DISCUSSION

MDMs have been accepted globally as the ‘gold standard’ for the management and care of cancer patients.^3^, ^16^ However, there is a need for research to further investigate the optimal structure and characteristics of MDMs.^10^ Whilst there are some international recommendations and guidelines for MDT functioning,^4^, ^14^ there are no universally standardised frameworks for how MDMs should be conducted. Furthermore, whilst existing guidelines provide a more formal and structured way to assess the quality of MDMs, the development of these guidelines such as those described by Taylor et al and Ottevanger, are not evidence driven. The quality criteria included in the guidelines, such as teamwork, clinical decision‐making, meeting organisation and logistics largely rely on consensus opinion, rather than drawing on robust evidence of their impact on the quality of MDM functioning.

A key challenge to the design of future studies is the breadth of proposed quality criteria to be examined as recommended by studies included in this review. Even though guidelines have been developed to identify areas for improvement in MDMs, further studies are required to robustly explore whether improvement in such areas will optimise MDM functioning. Until more conclusive data are generated, it will not be possible to develop an evidence‐based framework that can be adopted by cancer MDMs to standardise and inform best practice.

The streamlining of patient cases based on complexity was addressed in three studies.^16^, ^18^, ^35^ Streamlining patient cases are proposed as one way of prioritising cases for discussion at MDMs.^16^ However, despite proposing a potentially beneficial change to increase MDM efficiency, the implementation of streamlining has yet to be validated in an empirical study to assess whether streamlining improves the quality of decision‐making processes or, risks quality and safety of care where cases are not discussed. The MeDiC tool by Soukup et al may be a useful tool to identify complex cases and help facilitate such a study.^35^

Many of the studies included in this review focused on exploring factors that affect the quality of clinical decision‐making. Aside from ensuring that radiological and pathological information are present, the availability of information on patient's co‐morbidities and psychosocial history was also identified as being important to the clinical decision‐making process.^22^ Many MDMs were found to have limited information on patient co‐morbidities and psychosocial profile, which may suggest that current MDMs lack a patient‐centred focus when it comes to making treatment recommendations.^17^, ^36^ This is an area that needs to be further addressed to ensure that clinical decisions are individualised. One major limitation observed across all studies was that they focused only on the ability of MDM members to reach a clinical decision, as opposed to focusing on the quality of decisions made. Hence, assessing whether decisions made follow best‐practice clinical guidelines may be a more appropriate outcome measure for future studies.

Team dynamic was identified as one factor that impacts the quality of clinical decisions.^37^ Rowlands et al identified that discussions during MDMs were heavily dominated by doctors with limited input from nurses and allied health professionals.^26^ Whilst this study focused on a single lung cancer MDT at one hospital, similar observations were reported in another study.^20^ The dominance of discussion by and amongst doctors in MDMs can be more prominent when doctors also take on the role of the chairperson.^23^ This raises the question of whether having a doctor as MDM chairperson limits involvement and contribution of all team members. Evaluating nursing or MDM coordinator models of MDM leadership may offer opportunity for valuable future research.^23^ The skewness of MDM dynamic towards doctors appears to contradict the idea of a multidisciplinary meeting and may excessively narrow the focus of the clinical decision‐making suggesting that simply bringing together health professionals from different disciplines, may be insufficient to ensure comprehensive decision‐making processes.^26^ MDM team dynamic and communication is an area that warrants further research.

Currently, there is considerable heterogeneity across study designs and levels of evidence available to inform function and effectiveness of MDMs. Most papers included in this review used either qualitative, mixed methods or cross‐sectional approaches. Data generated were largely descriptive in nature and could therefore only provide summary descriptions of strengths and weaknesses of current MDMs. Of the 25 papers reviewed, only one^12^ was a prospective interventional study, which set out to establish the impact of a new intervention on the decision‐making process within an MDM. The lack of more robust empirical studies in the current literature suggests that there should be a shift away from descriptive studies, and a stronger focus on experimental studies to establish evidence‐based interventions to improve the effectiveness of MDMs.

This literature review has the following limitations: Papers reviewed are from different countries with different health systems, limiting transferability of findings across different health systems. Many studies focused on the MDT of a single disease site, further limiting generalisability of findings to MDMs for other cancer types. Furthermore, due to the heterogeneity of the studies, it was not possible to undertake a meaningful risk of bias assessment and as such not possible to make definitive statements about the quality of evidence currently available.

## 5 CONCLUSIONS

MDMs continue to be a central component of cancer care delivery worldwide. Whilst evidence regarding the impact of MDMs on cancer survival is equivocal, this narrative review was able to provide a comprehensive summary of factors that may influence the quality of MDMs. The large body of evidence on MDMs focuses predominantly on developing tools to identify barriers to conducting an optimal MDM. Factors such as the quality of teamwork, leadership roles and availability of clinical information have all shown to impact on the decision‐making process of MDMs, a common outcome measure of MDM effectiveness. There is scope for future research to utilise more robust interventional studies to explore the impact of these factors in greater depth. Interventions to encourage greater clinician engagement as well as enabling greater contribution by allied health professionals or nurses may allow for a more collaborative and holistic approach to clinical decision‐making. Additionally, the presence of core decision‐enabling members such as radiologists, better planning to ensure that all patient clinical information is available and streamlining more complex cases were identified as important considerations with potential to improve the quality and implementation of treatment recommendations at MDMs. The findings of this review contribute to the development of an evidence‐driven framework to help standardise the way in which oncology MDMs are conducted.

## COMPETING INTERESTS

The authors have declared that no competing interests exist.

## Supporting information

Data S1
Table S1

## Data Availability

The data that supports the findings of this study are available in the supplementary material of this article.
