# Supplementary material for: Optimising the quality of multidisciplinary team meetings: A narrative review
Source: Cancer Med. 2022 Mar 7;11(9):1965–71. doi: 10.1002/cam4.4432 (PMC9089217; doi:10.1002/cam4.4432)
Supplement: Supplementary file 1 — Data S1 Table S1 [file CAM4-11-1965-s001.docx]

**Supplementary Data 1:** Medline Search Strategy

Database: Ovid MEDLINE(R) and Epub Ahead of Print, In-Process & Other Non-Indexed Citations and Daily <1946 to September 04, 2020>

1 Patient Care Team/st (3236)

2 (multidisciplinary adj3 team*).tw. (21206)

3 1 or 2 (24142)

4 interdisciplinary communication/ (17276)

5 leadership/ (41270)

6 decision-making/ (95666)

7 (team-work* or decision-making* or communicat*).tw. (423200)

8 4 or 5 or 6 or 7 (532297)

9 3 and 8 (4629)

10 (structur* or logistic* or infrastructur* or organi?ation* or framework* or guideline*).tw. (3416756)

11 guideline/ (16292)

12 10 or 11 (3425207)

13 9 and 12 (1749)

14 quality improvement/ (25122)

15 (effect* or efficien* or effica* or eval* or quality).tw. (10446007)

16 14 or 15 (10453302)

17 13 and 16 (1163)

18 exp Medical Oncology/ (23207)

19 exp Neoplasms/ (3356577)

20 (cancer* or tumo?r* or oncolog*).tw. (2868044)

21 18 or 19 or 20 (4222581)

22 17 and 21 (247)

23 (meeting* or board*).tw. (205435)

24 22 and 23 (92)

Supplementary Table 1: Characteristics and key findings of the included studies

| Author  (Year) | Aim | Evaluation Methods | Key Findings | Limitations |
| --- | --- | --- | --- | --- |
| Lamb et al.^20^  (2011) | To assess the quality of information presentation and MDT members' contribution to decision-making | A surgeon uses an observational tool to assess 5 MDTs  Members at the MDTs completed a self-assessment survey tool  The surgeon’s assessment and the teams' self-assessment were compared | Case histories and radiological information were best presented  Patients' views and comorbidities/psychosocial issues were poorly addressed  Surgeons and oncologists made the greatest contributions while nurses and MDT coordinators made little contributions to the discussions | Small sample size  Only urology MDTs - may not be generalisable to other tumour type MDTs |
| Lamb et al.^29^  (2011) | To construct a robust tool for scientific assessment of MDT performance | A tool was adapted from a pre-existing tool that assesses team performance  This new tool was used in five MDT meetings to assess validity and reliability | Contributions of surgeons, chair's effectiveness, presentation of case history and radiological information were rated above average  The interobserver agreement was high for presentation of radiological information, and contribution of clinical MDT members. | Small sample size and unrepresentative of MDTs in general  Mainly representative of urological MDTs  There is ambiguity in how silence in a meeting is interpreted by observers using the tool |
| Lamb et al.^21^  (2011) | To develop an intervention to standardise and improve the quality of case discussion and clinical decision making in MDT meetings. | The checklist was developed in 3 phases  Participants were surveyed for their opinion about using a checklist for MDMs  Participants' feedback were taken into account for the final version of the checklist | More than half of participants agreed that the checklist should be used as a memory aid to guide discussion and help prepare cases.  Nurses were the main group that agreed that the checklist would allow them to contribute more to the MDTs | The majority of the participants practiced only in urology  Only 4 professional groups were included |
| Patkar et al.^30^  (2012) | To develop and implement a novel clinical decision support (CDS) platform for breast cancer MDT and evaluate the concordance between the CDS suggestions and MDT recommendations | A computerised decision support system, MATE, was developed and the suggested treatment recommendations made by this CDS was compared with guideline recommendations made by the breast MDT members | The level of concordance between MATE recommendations and the actual MDT decisions was 93.2%  MATE identified 61% more patients who were potentially eligible for recruitment into clinical trials than the MDT alone | The MATE was only trialled at breast MDMs and hence may not be generalisable to all MDMs  Results needs to be validated via a randomised study design |
| Taylor et al.^32^ (2012) | To develop and test an MDT self-assessment tool | Questionnaires underwent statistical analysis and semi-structured interviews were coded and analysed thematically | The content of TEAM questionnaire was valid  The domain-scales for the items in the TEAM questionnaire had acceptable internal consistency and good item discrimination  Interviewed team members of MDTs were positive about the TEAM tool | TEAM was only tested by local cancer teams treating common tumours - may need changes to accommodate for different team structures or processes  Just over half of the team members of the MDTs completed the survey - limited participation |
| Taylor et al.^13^  (2012) | To develop quality criteria for assessment of characteristics observable in MDMs and test inter-rater reliability and feasibility | The MDT-OARS was piloted with ten bowel MDMs and feasibility was estimated by collecting data on the time to complete the assessments  Inter-rater reliability was assessed by comparing the ratings of the research team with two independent observers | The MDT-OARS was acceptable to teams and feasible to implement  There was variability in quality of the MDMs across the 10 MDTs  Inter-rater reliability was achieved across all items on the tool except two characteristics of effective teamworking | Small pilot study sample size  The MDT-OARS needs to be tested with other tumour types and more teams |
| Rowlands et al.^26^  (2013) | To explore how patient information is communicated between health professionals within a multidisciplinary lung cancer team | Data from the semi-structured interviews analysed thematically using the ground theory | There is limited communication between doctors and nurses and allied health professionals  Doctors were more likely to communicate with other doctors with little input coming from nurses and allied health professionals  Most of the communication was dominated by doctors | Only focused on a lung cancer team in one hospital |
| Lamb et al.^37^  (2013) | To explore the views of MDT members regarding contribution to the MDT, representation of patients' views and dealing with disagreements in MDT meetings | Free text responses of 6 questions in the 2009 National Cancer Action Team Survey were analysed thematically | Non-technical skills including mutual respect and understanding, enthusiasm and positivity, good communication, sharing of common goals, good leadership, patient centredness were considered important  Lack of knowledge of patient's views, lack of personal contact with patient prevent recommendations from being implemented | Responses were subjective opinions and self-reported  A snowballing method was used to recruit participants |
| Lamb et al.^12^ (2013) | To develop and evaluate a multicomponent intervention designed to improve MDM's ability to reach treatment decisions | MTB coordinator objectively recorded whether a treatment decision during each MDM  Quality of teamwork and quality of information presentation was measured using the MODe tool | With the interventions there were improvements in the number of decisions reached, global information quality and global teamwork score.  There was a decrease on meeting decision and time spent on each patient suggestive of increased time efficiency.  Main reasons for failure to reach a decision were due to inadequate pathology, radiology, inappropriate patient referrals. | There were no controls  Presence of the Hawthorne effect where teams may have improved just by being observed |
| Lamb et al.^22^  (2013) | To assess the relationship between the quality of the presented information, contribution to discussion of team members, team size, case positioning within the meeting, and timing and ability to reach clinical decisions | Each MDT meeting was observed and evaluated using the MDT-MODe tool | Clinical decisions were reached in 85% of cases discussed  The reasons why a decision was not reached were due to insufficient radiological information, inadequate pathological information, inappropriate referral to the MDT, lack of clinical notes and non-attendance of team-members  Cases positioned towards the beginning of the meeting were associated with higher scores | Sample of MDT come from a single hospital  The study focuses on urology MDTs - might not be applicable to all tumour types  Can't explain the causation between the characteristics discussed |
| Ottevanger et al.^10^  (2013) | To develop a guideline with quality criteria for an optimal structure and functioning of a multidisciplinary team meeting and to assess to what extent the Dutch MDMs complied with these criteria | The guideline with quality criteria for effective MDMs was developed  Evaluation of compliance with the quality criteria was done by interviewing the chairs of various MDMs | The guideline for effective MDMs had 5 main domains: organisation of MDMs, membership of the MDM and roles and responsibilities of members, the meeting itself, documentation of meeting recommendations  100% attendance of core members was never achieved and the role of the chair needs improvement. | The guideline developed is not evidence-driven  There was observation bias as there was difficulty in making reliable evaluations during videoconferences where certain aspects were unclear  Inter-observer variation |
| Jalil et al.^17^ (2013) | To investigate the views of expert urology and gastro-intestinal cancer service providers in relation to effectiveness of their MDTs in reaching a recommendation for patients and implementing this recommendation | Interviews were audio-taped and transcribed verbatim  A coding framework of emerged common themes was developed by two trained clinical researchers to analyse all interviews independently | Video conferencing can interfere with the meeting and impede teamwork and team decision-making  Lack of information on patients' comorbid and social history, patients' preferences, and investigations results obstructs decision-making  Absence of a core team-member would result in delayed decisions or result in inappropriate treatment plan  Time-pressure of meetings may results in patients being deferred to the following week's meeting and a delay in their management plan | Small sample size  Not representative all tumour types - only urology and GI surgery MDTs were interviewed  Views may not be generalisable to other MDTs |
| Taylor et al.^36^ (2014) | To examine cancer MDT members' views on how best to ensure that patients are meaningfully involved in decision-making about the care in the context of the MDT and MDT meetings | Interviews were recorded digitally, transcribed and analysed thematically by two experienced qualitative researchers independently | Patients had limited opportunities provide input to or influence the decision making process in MDMs.  There is potential influence of patient knowledge about medicine on their understanding or recall of information about MDTs and MDMs  One of the benefits of informing patients about MDTs and MDMs is to allow for reassurance | Small sample size  Only representative of a small subgroup of cancer Patients and MDT members |
| Harris et al.^33^  (2014) | To develop a structured peer observational assessment tool and explore its usability and feasibility at MDMs | Meetings were observed by chosen peer observers using the prototype observational tool  Semi-structured telephone interviews were conducted  Interviews were analysed using thematic content analysis | The prototype observational tool was easy to use however the rating scale was too broad and non-specific  Most peer observer (12/19) stated that the tool was feasible  Most peer observers and MDT members agreed that observational feedback was useful and could inform improvement in MDT performance | A subjective study as most of the results were based on the opinions of the peer observers and MDT members  Possible Hawthorn effect in the performance of MDMs  It didn't explore whether feedback from the observational tool actually resulted in change or improvement of MDT workings  The results may not be generalisable across other MDTs |
| Soukup et al.^23^ (2016) | To explore the underlying structure of decision-making process and examine how it relates to a team’s ability reach a decision | Secondary analysis of an existing database containing observational data on the ability of MTBs to make a treatment decision for 1045 cancer patient cases  These MTBs were evaluated using the MDT-MODe | Holistic and clinical inputs, radiology and pathology contributed the most to the probability of the team reaching a treatment decision  Surgeons who chaired MDMs and also provide input to the case reviews exhibited dual-task interference where as the surgeon's input to case reviews increased, the chair's input decreased, affecting the coordination of the meeting | Observer bias  MDT-MODe may not accurately evaluate the complex role of the MDT chairperson and coordinator  Confounding effects of different tumour types  Data was derived from different institutions and so the different team culture, values, beliefs and attitudes could influence results |
| Harris et al.^34^  (2016) | To develop a tool for independent observational assessment of cancer multidisciplinary team meetings | Criterion validity was established if observers were able to discriminate between optimal and suboptimal performance  Descriptive statistics were used to assess performance scores of each domain within the MDT-MOT | The MDT-MOT can reliably discriminate between better and worse MDT performance  There was diversity in performance between MDTs regarding leadership, chairing of the meeting, organisation, administration and clinical decision making processes | Re-enacted meetings were used for study 1 which were shorter excerpts and may not be a good representation of real MDMs  Small sample in both Study 1 and Study 2 |
| Soukup et al.^2^ (2016) | To evaluate how different elements of the decision making process affect the teams' ability to reach a decision on first case review | Secondary analysis of an existing database containing observational data on the ability of MTBs to make a treatment decision for 1045 cancer patient cases  These MTBs were evaluated using the MDT-MODe | Positive significant predictors of treatment decisions were patient psychosocial information, inputs to case reviews by radiologists, pathologists, surgeons and oncologists  Negative significant predictors of treatment decisions were patients' comorbidities and nurses input | Observer bias  Results may not be generalisable as data was mainly obtained from the English National Health Service |
| Hahlweg et al.^24^  (2017) | To systematically assess the quality of decision-making processes at MDMs | An adaptation of Lamb and colleagues’ MDT-MODe was used to assess the quality of clinical treatment recommendation process in MDMs | Cancer specific medical information was presented with the highest quality  Patient views, psychosocial information and information on comorbidities were presented with lower quality  Higher level of medical and treatment uncertainty was associated with a higher probability of giving more than one treatment recommendation  Quality of MDM chair behaviour was low | This study might not be generalisable to other institutions and countries  Observations were carried out by psychologists, limiting the validity of assessments regarding specialist medical issues |
| Peckham et al.^18^  (2018) | To determine the contributions of an oncology nurse navigator related to physician adherence to guidelines and streamlined patient care in a lung cancer tumour board | Chart review of a lung cancer tumour board performed for 18 months pre-implementation and 18 months post-implementation of intervention | There was an increase in the following implementation of the intervention:   - Diagnosis of early-stage non-small cell lung cancer following implementation of intervention - Patients seeing a pulmonologist prior to treatment - Patients completing pulmonary function tests respectively - Patients receiving PET- CT scans prior to the start of treatment | Study was only conduced at one facility and had a limited sample size of 83  Most retrospective data collected during a limited time period of 18 months |
| Lumenta et al.^11^  (2019) | To use a published tool as a template for the development of a teamwork assessment tool and to evaluate the feasibility of the tool | An adapted version of the MDT-MODe was developed  The feasibility study was carried out using the MDT-MODe tool to evaluate the performance of the tumour board meetings | In general there were high agreements amongst the non-clinical and clinical observers  Non-clinical observers found it hard to assess aspects regarding presentation of the case history and patient data, laboratory results, pathology, X-ray and involvement of all team members. | Study was conducted at a single institution which might not be representative of other MDT meetings  Lack of representation of all professional groups in choice of observer |
| Rosell et al.^5^  (2019) | To use the MDT-MODe and MDT-MOT to assess contributions from various health professionals during MDMs | Participants' responses to questionnaire was evaluated based on the Likert-type scale  MDT-MODe and MDT-MOT data were statistically analysed | MDMs were rated favourably for their ability to develop individual competence and team competence  MDT- MOT results: High scores were obtained for clinical decision making process, teamworking and culture, technology and equipment, physical environment and leadership and chairing. Low scores applied to patient-centred care, organisation, administration and post-meeting coordination  MDT-MODe results: Case information and case discussion by the chair, surgeons, and oncologists scored high and low scores applied to information on psychosocial aspect and patient's views | Low response rate to questionnaires of 52%  Observational tools does not account for the scoring of individual specialists and do not take into account features that may not be relevant to the MDMs  There were minimal responses from nurses and coordinators. Hence, subgroup analysis was not possible |
| Hoinville et al.^16^  (2019) | To obtain an understanding of MDT members' opinions about streamlining patient discussions at MDMs | Qualitative analysis of percentage of respondents who agreed, disagreed or had no opinion about each of the 12 statements relating to streamlining was examined  Responses to free-text questions were analysed by inductive coding | Whilst there is agreement for streamlining of MDM discussions (focus on more complex cases), this may not be appropriate for all MDTs and there was a lack of consensus about the methods by which streamlining could be achieved. | Possibility of non-response bias  Participants has varying understandings of the term 'streamlining' which may have skewed their responses  Questions used in the questionnaire were ambiguous and hence responses were hard to interpret  There was not an equal representation of some MDTs for some tumour types |
| Soukup et al.^27^ (2020) | To test the functional perspective of group decision making in cancer MDTs | Assessments were conducted from video recordings using three validated observation tools as shown below:  MDT-MODe  Bales Interaction Process Analysis  MEDiC | Positive predictors of decision-making quality were asking questions, providing answers, team size, gender balance and clinical complexity  Negative predictors of decision-making quality were negative socioemotional reactions, gender imbalance, logistical issues, time-workload pressures and time spent in the meeting | Observer bias - Hawthorne effect  The safety and clinical implications of the study are not explored  May not be generalisable to other cancers as study only represents the most common cancers within the UK NHS |
| Neri et al.^19^ (2020) | To assess the quality and amount of radiologists in MTBs, their role in it and related issues | Responses from a questionnaire consisting of 15 questions were collected and statistically analysed | Few radiologists review imaging studies prior to the MTB meetings due to poor quality imaging studies and lack of time  Attendance of radiologists at meetings changes the diagnostic strategy or refine the therapeutic decisions  Only 58.8% of the radiologists were mandated to attend MTB meetings while the remainder had the option to choose | Selection bias due to surveying only one group  High prevalence of participants from Italy |
| Soukup et al.^35^  (2020) | To develop and validate an evidence-based and expert-derived tool for assessing complexity of a cancer patient's case - the MeDiC tool | The MeDiC tool was developed and evaluated for feasibility, reliability and validity | Cases that obtain higher MeDiC scores take significantly longer time to discuss and make a treatment plan for within MTBs | Only developed and tested within the UK's fully MTB-driven cancer care system  The tool was tested in real time when it should be used to help with the preparation and streamlining of patient cases in advance  The expert review team were mainly surgeons and psychologists hence lacking insight from other experts |
| Ghazal Asswad et al.^25^ (2020) | Audit the MDT decision-making process in relationship to national guidelines and implementing of MDM recommendations | Retrospective analysis of MDT meetings over a 12-month period | The majority of MDT decisions followed guidelines and any discordant decisions were justifiable | Results of a single UK tertiary hospital |
| Pluyter et al.^31^ (2020) | Development and user testing of CDS system that maximises the ability for the MDM to make high quality clinical recommendations | CDS systems was tested MTB meeting for primary diagnosis and treatment selection for primary lung cancer cases (n=8). | CDS system supported the team in self-correcting capacity for accurate diagnosis and TNM classification | CDS tested in one hospital |
| Rosell et al.^28^ (2020) | To determine the enabling factors and barriers for national, virtual MDMs for rare cancers | Responses from a questionnaire were collected and qualitative analysis was performed | National virtual MDMs provide support in decision-making, strengthen collaborations and professional networks, and develop individual and team-related competence. Barriers are uncertain assignments and responsibilities and suboptimal collaboration between hospitals, as well as, suboptimal consideration of patient-related information, time constraints and non-attendance from core members | A response rate of 52%, no possibility for analysis of non-respondents, and the free-text questions are not matched to quantitative data |
| Soukup et al.^15^ (2021) | A cross-sectional observational study of MDM fidelity assessed against stages of group decision-making and adherence to cancer guidelines | Prospective cross-sectional observational study across three UK hospitals | Cancer case-reviews in MDM are not entirely MDT-driven, with more than half of the case-reviews not adhering to the cancer guidelines, and just over 10% not adhering to the group decision-making framework | A limited number of cases was included (N=24) and, as an observational study, data can be influenced by the Hawthorne effect |

**Abbreviations:** CDS, clinical decision support; CT, computerised tomography; GI, gastrointestinal; MATE, Multidisciplinary meeting Assistant and Treatment sElector; MDM, multidisciplinary team meeting; MDT, multidisciplinary team; MDT-MODe, MDT Metric of Decision Making; MDT-MOT, MDT Meeting Observational Tool; MDT-OARS, MDT Observational Assessment Rating Scale; MDT-QuIC, MDT Quality Improvement Checklist; MEDiC, Measure of case- Discussion Complexity; MTB, multidisciplinary tumour board; NHS, National Health Service; PET, positron emission tomography; TEAM, Team Evaluation and Assessment Measure; UK, United Kingdom.
